# Self-management and related factors in patients with systemic lupus erythematosus: a cross-sectional study

**DOI:** 10.3389/fmed.2025.1700387

**Published:** 2026-01-02

**Authors:** Wei Xu, Yan Chen, Xueying Shang, Yan Zhao, Jingru Liu, Jun Li, Bei Zhang, Ping Wang, Xin Lu

**Affiliations:** 1 Department of Gastrointestinal Surgery, The First Affiliated Hospital, College of Clinical Medicine, Henan University of Science and Technology, Luoyang, Henan, China; 2 Henan Medical Key Laboratory of Gastrointestinal Microecology and Hepatology, Department of Gastroenterology, The First Affiliated Hospital, College of Clinical Medicine, Henan University of Science and Technology, Luoyang, Henan, China; 3 Department of Public Health, School of Medicine, Henan University of Science and Technology, Luoyang, Henan, China

**Keywords:** systemic lupus erythematosus, self-management ability, self-efficacy, family care index, medical response

## Abstract

**Objective:**

To assess the self-management status of patients with SLE and identify the factors that influence it.

**Background:**

Systemic lupus erythematosus (SLE) is a chronic, relapsing autoimmune disease that requires lifelong management. Effective self-management is crucial for reducing disease activity, economic burden, and improving quality of life. However, research on SLE-specific self-management tools and influencing factors remains limited.

**Design:**

A cross-sectional study was conducted from March to September 2024, enrolling 370 SLE patients from three tertiary hospitals in Henan Province. Data were collected using validated scales, including self-management, self-efficacy, illness perception, coping styles, family support, and social support. Univariate analyses, correlation analyses, and multiple linear regression were performed.

**Methods:**

A cross-sectional study was conducted from March to September 2024, enrolling 370 SLE patients from three tertiary hospitals in Henan Province. Data were collected using validated scales, including self-management, self-efficacy, illness perception, coping styles, family support, and social support. Univariate analyses, correlation analyses, and multiple linear regression were performed. Multicollinearity was assessed using variance inflation factors (VIFs) and tolerance values.

**Results:**

Among the 370 SLE patients, the mean age was 38.16 ± 12.81 years; the mean duration of illness was 4.65 ± 3.18 years; Regarding disease activity, 134 SLE patients (36.2%) had no or minimal activity, 116 (31.4%) had mild activity, and 120 (32.4%) had moderate-to-high activity. The mean self-management score was 59.06 ± 16.75, with sub-scores for medication being 9.91 ± 3.45, disease surveillance (10.01 ± 3.56), daily life (29.40 ± 9.35), and reproductive health (9.74 ± 3.84). The key influencing factors included disease activity, education level, marital status, self-efficacy, illness perception, coping styles, family support, and social support, explaining 52.4% of variance.

**Conclusion:**

Tailored interventions should address SLE patients’ unique needs by enhancing self-efficacy, optimizing illness perception, promoting positive coping, and strengthening social and family support. This approach can improve self-management, reduce disease burden, and enhance the quality of life.

## Introduction

Systemic lupus erythematosus (SLE) is a complex autoimmune disease with an unclear etiology that affects multiple systems (1). It is characterized by severe conditions, high recurrence rates, and low long-term remission rates, and it is commonly found in women, particularly those of child-bearing age (2, 3). Studies show that the prevalence of SLE in China is between 97.5 and 100 per 100,000, with an annual incidence rate of 3.1 per 100,000, and it shows an increasing trend (4, 5). The clinical manifestations of SLE are complex and varied, ranging from mild skin and joint involvement to severe organ failure and obstetric complications, significantly reducing the quality of life for patients and imposing a considerable financial burden on them (6).

With the development of treatment methods, the survival rate of SLE patients has significantly improved, but the physical and mental burden as well as role conflict issues arising from long-term treatment remain prominent (7–9). The self-management ability of patients has become a key factor in maintaining disease stability and improving quality of life. Self-management for SLE patients refers to their ability to actively participate in managing, monitoring, and guiding their own behavior during the illness (10). Research has shown that effective self-management can not only improve symptom control for SLE patients and enhance medication adherence but also reduce psychological stress and economic burden (11–13). At the current stage, research on self-management of chronic diseases is becoming increasingly active, but empirical studies on self-management among SLE patients are still lacking, particularly in the systematic exploration of influencing factors, depth of theoretical guidance, and comprehensiveness of measurement dimensions.

Currently, the tools for assessing active participation in care and disease management among SLE patients primarily use the patient activation measure (PAM) (5), which is applicable to all chronic disease populations and has universality, but lacks SLE specificity. Chinese scholar Cui Xiaowen developed a self-management assessment scale for lupus patients that is suitable for the characteristics of Chinese SLE patients based on the Chinese cultural context. This scale has good reliability and validity, a clear structural dimension, and covers multiple aspects—including medication management, disease monitoring management, daily life management, and reproductive health management—providing a comprehensive and scientific assessment of the current status and characteristics of SLE patients’ self-management behaviors (14).

Social ecological system theory posits that individual behavior is influenced by the social environment and their interactions, as well as the interplay and mutual influence between the interconnected micro-, meso-, and macro-level systems of individual development (15–17). Existing research indicates that studies on the influencing factors of SLE self-management primarily focus on the microsystem—such as demographic factors, psychological factors, and disease information—lacking examination of the factors across multiple systems. This study, based on the social ecological system theory, uses a locally developed SLE self-management-specific scale with good reliability and validity for a cross-sectional survey. It explores the current self-management status of patients with systemic lupus erythematosus (SLE) from the micro (self-efficacy, disease perception, coping styles), meso (family care), and macro (social support) systems, and analyzes the factors influencing self-management in order to inform SLE health management strategies.

## Methods

### Study design and participants

This study adopted a convenience sampling method to select hospitalized patients with systemic lupus erythematosus (SLE) in the rheumatology and immunology departments of three tertiary hospitals in Henan Province from March 2024 to September 2024 as the research subjects. Inclusion criteria were as follows: (1) a clinical diagnosis of SLE meeting the revised classification criteria of the American College of Rheumatology (ACR) (18); (2) a confirmed diagnosis of SLE for at least 1 month; (3) age 18 years or older; (4) clear consciousness, capable of understanding and expressing, able to complete the questionnaire independently or under the guidance of the investigator; (5) awareness of their condition and willingness to participate in this study. Exclusion criteria were as follows: (1) the presence of other severe life-threatening diseases; (2) mental disorders.

This study has 28 independent variables. According to the requirements, the sample size should be at least 5–10 times the number of independent variables (19), which amounts to 140–280 cases. Considering a 20% non-response rate, the sample size is 175–350 cases. To ensure the reliability of the results, a total of 380 questionnaires were distributed in this study, with 370 valid questionnaires returned, resulting in an effective response rate of 97.4%. This study has been approved by the Ethics Committee of the First Affiliated Hospital of Henan University of Science and Technology (2023-03-K0049).

### Measures

#### General information questionnaire

Based on a literature review and discussions with the research team, a general data information questionnaire was developed. It included gender, age, work status, SLE disease activity, duration of illness, educational level, marital status, per capita monthly family income, family residence, medical expense payment method, whether biological agents are used, and presence of complications, etc. Among them, the SLE disease activity is assessed using the currently most widely used Systemic Lupus Erythematosus Disease Activity Index-2000 (SLEDAI-2000), which consists of 24 items, with each item having two parts of responses: “no such condition” and “has this condition” (20). Responses to items 1 to 8 that are “Yes” score 8 points each; otherwise, 0 points. Responses to items 9 to 14 that are “Yes” score 4 points each; otherwise, 0 points. Responses to items 15 to 21 that are “Yes” score 2 points each; otherwise, 0 points. Responses to items 22 to 24 that are “Yes” score 1 point each; otherwise, 0 points. The final total score indicates the activity level of SLE patients: A total score of 0 to 4 indicates minimal activity; a score of 5 to 9 indicates mild activity; a score of 10 to 14 indicates moderate activity; and a total score of 15 or more indicates severe activity.

#### SLE Self-Management Scale

The SLE Self-Management Scale was developed by Cui Xiaowen et al. (14) for the Chinese population and is used to assess the self-management ability of SLE patients. The scale consists of 25 items across four dimensions: medication management, disease monitoring management, daily life management, and reproductive health management. It employs a five-point Likert scale, with response options ranging from “never” to “always” (scored 1 to 5). Higher scores indicate better self-management ability in patients. Scores on the SLE Self-Management Scale are interpreted based on the total score and subscale scores. Higher total scores indicate better overall self-management abilities. Each of the four dimensions—involving medication management, disease monitoring management, daily life management, and reproductive health management—has its own subscale score, which reflects the patient’s proficiency in that specific area of self-management. The scale provides subscale categories for each dimension, allowing for a more nuanced understanding of a patient’s strengths and weaknesses in managing different aspects of their disease. These subscales help identify the areas where targeted interventions may be necessary. No normalization of the scores was performed in this study. Raw scores were used for analysis, with means and standard deviations reported for both total scale scores and the subscale scores. In future studies, normalization could be considered if needed to adjust for population-level variations.

The Cronbach’s *α* coefficient of the scale is 0.888, the split-half reliability is 0.742, the test–retest reliability is 0.846, and the content validity index is 0.980.

#### Chronic Disease Self-Efficacy Scale

The Chronic Disease Self-Efficacy Scale (SEMCD) was developed and simplified to a six-item version by Lorig et al. (21) at Stanford University. In this study, the Chinese version of the Chronic Disease Management Self-Efficacy Scale (C-SEMCD) was used for investigation. This scale consists of six items, with the first four reflecting self-efficacy in symptom management and the last two reflecting self-efficacy in disease management. Each item is rated on a scale of 1 to 10, indicating increasing levels of confidence, from “no confidence” to “complete confidence.” The total score is the average of the six items, with a score less than or equal to 4.0 indicating low level, scores from 4.0 to 7.9 indicating moderate level, and scores greater than or equal to 8.0 indicating high level. The Cronbach’s *α* coefficient for the Chinese version of the Chronic Disease Management Self-Efficacy Scale ranges from 0.88 to 0.95, indicating good internal consistency.

#### Brief Illness Perception Questionnaire

The Brief Illness Perception Questionnaire (BIPQ) was developed by Broadbent et al. and was revised in Chinese by Ma et al. (22, 23). This scale includes cognitive dimensions (items 1, 2, 3, 4, and 5), emotional dimensions (items 6 and 8), and understanding ability dimensions (item 7), with item 9 being an open-ended question. Each item is scored from 0 to 10, with a total score range of 0 to 80. The reverse scored items are items 3, 4, and 7, where a higher score indicates a more severe negative perception of the disease threat. The Cronbach’s *α* coefficient for the Chinese version of the BIPQ is 0.77.

#### Medical Coping Styles Questionnaire

The Medical Coping Styles Questionnaire (MCMQ), developed by Feifel et al. (24), was revised into a Chinese version by Shen Xiaohong and others. It mainly measures the coping styles individuals adopt when faced with illness, including three dimensions: confrontation, avoidance, and submission, with a total of 20 items. A four-point Likert scoring method is used, where each item is scored from 1 to 4 in ascending order, resulting in a total score range of 20 to 80 points. The dimension with the highest score indicates that the individual is more inclined to use that coping style to deal with their current health condition. The Cronbach’s alpha coefficients for the three subscales are 0.69, 0.60, and 0.76, with test–retest reliability coefficients of 0.64, 0.85, and 0.67, respectively (25). The MCMQ has good reliability and validity, with concise items that are easy to measure, and it is now widely used in China.

#### Family Apgar Index

The Family APGAR Index questionnaire, developed by Smilkstein et al. (26) and revised by Zhao et al. (27), allows for a subjective and quantitative assessment of family functioning in a short amount of time. It includes five dimensions: adaptability, partnership, growth, affection, and intimacy, with a total of five items. A three-point Likert scale is used, where scores of 0–2 represent “very rarely,” “sometimes,” and “often,” respectively. The scoring range of the questionnaire is from 0 to 10 points, where 0–3 points indicate severe family dysfunction, 4–6 points indicate moderate dysfunction, and 7–10 points denote good family functioning. The Cronbach’s *α* coefficient of this scale is 0.813, and the split-half reliability is 0.703, indicating good reliability and validity.

#### Social Support Rate Scale

The Social Support Rate Scale (SSRS), developed specifically for Chinese people by Shao et al. (28) in 1986, has been widely used in China to measure individual social support levels. The scale includes three dimensions: subjective support, objective support, and support utilization, comprising a total of 10 items. Each dimension uses a four-point scoring system, where answers “no sources” for items 6 and 7 score 0 points, and “the following sources” score points according to the number of sources. The total score is the sum of the item scores, ranging from 12 to 66 points: 12–22 points indicate low level, 23–43 points indicate moderate level, and 44–66 points indicate high level. A higher score indicates better social support levels for the individual. The overall consistency reliability of the scale is 0.92, and the test–retest reliability is between 0.89 and 0.94.

### Data collection

Nurses trained uniformly by researchers were appointed as investigators to conduct standardized questionnaire surveys on patients who met the inclusion criteria. Prior to the formal survey, a pre-survey was conducted to check the logic and comprehensibility of the questionnaire, and improvements were made based on the results of the pre-survey. When distributing the questionnaires, investigators explained the purpose, methods, and important notes of the study to the participants and obtained informed consent before they filled out the questionnaires independently. A uniform guiding script was used to address any questions raised by the subjects. After the questionnaire was completed, it was collected on-site, checked by two people, and if there were any missing items or incomplete sections, data were supplemented and completed on the spot. A total of 380 questionnaires were distributed, with 370 valid questionnaires collected, resulting in a valid response rate of 97.4%.

### Data analysis

SPSS 25.0 statistical software was used for data verification, entry, and analysis. To ensure data integrity, patients with any missing information (e.g., incomplete scale responses, missing clinical information) were excluded from the analysis. Measurement data that meets the normal distribution is expressed as mean plus/minus standard deviation, while data that does not meet the normal distribution is expressed as median and quartiles. Count data are described using frequency and percentage. T-tests, ANOVA, or Chi-square tests are used for univariate analysis of self-management in SLE patients. Pearson correlation analysis is used. Multiple linear regression is employed for multifactor analysis of self-management. During the regression analysis, the variance homogeneity of residuals was evaluated by analyzing the distribution of standardized predicted values of residuals and scatter plots. The normality of residuals was tested by analyzing the histogram of standardized residuals. The independence of residuals was verified using the Durbin–Watson statistical test. Multicollinearity diagnosis was conducted by determination of the variance inflation factor (VIF) and tolerance. A *p*-value of <0.05 is considered statistically significant.

## Results

### General characteristics of the study subjects

Among 370 SLE patients, ages ranged from 18 to 80 years, with a mean age of 38.16 ± 12.81 years. Specifically, 106 patients (28.6%) were aged 18–30 years, 136 (36.8%) were aged 31–40 years, 106 (28.6%) were aged 41–60 years, and 22 patients (5.9%) were older than 60 years. There were 338 female patients (91.4%) and 32 male patients (8.6%). The duration of illness ranged from 1 to 12 years, with an average duration of 4.65 ± 3.18 years. Of these patients, 286 (77.3%) had a disease duration of less than 5 years, 64 (17.3%) had a duration of 5–10 years, and 20 (5.4%) had a duration of more than 10 years. In terms of disease activity, 134 patients (36.2%) had basically no activity, 116 patients (31.4%) had mild activity, and 120 patients (32.4%) had moderate-to-high activity. In addition, 254 patients (68.6%) had complications, while 116 patients (31.4%) had no complications. Regarding the use of biologics, 256 patients (69.2%) used biologics and 114 patients (30.8%) did not, as shown in Table 1.

**Table 1:** General characteristics of the study subjects.

Items	*n* (%)
Gender	
Male	32 (8.6)
Female	338 (91.4)
Age (years)	
18–30	106 (28.6)
31–40	136 (36.8)
41–60	106 (28.6)
>60	22 (5.9)
Duration of illness (years)	
<5	286 (77.3)
5–10	64 (17.3)
>10	20 (5.4)
Complications	
No	116 (31.4)
Yes	254 (68.6)
Disease activity	
Basically, no activity	134 (36.2)
Light activity	116 (31.4)
Moderate-to-high activity	120 (32.4)
Work situation	
Employed	90 (24.3)
Unemployed	158 (42.7)
Freelancing	122 (33.0)
Educational level	
Elementary school and below	56 (15.1)
Junior high school	98 (26.5)
High school or vocational school	72 (19.5)
Junior college	66 (17.8)
Bachelor’s degree or above	78 (21.1)
Marital status	
Married	239 (64.6)
Unmarried	93 (25.1)
Others	38 (10.3)
Monthly household income (RMB, Yuan)	
≤1,000	50 (13.5)
1,000–1999	80 (21.6)
2000–2,999	74 (20.0)
3,000–3,999	64 (17.3)
≥4,000	102 (27.6)
Healthcare insurance methods	
Employee Medical Insurance	194 (52.4)
Resident Medical Insurance	132 (35.7)
Others	44 (11.9)
Family residence	
City	192 (51.9)
Rural	104 (28.1)
County seat	74 (20.0)
Biologics	
Use	256 (69.2)
Not used	114 (30.8)

### Scores for self-management and items

The average self-management score among SLE patients was 59.06 ± 16.75. The mean entry scores across dimensions were as follows: daily life management (2.94 ± 0.94), disease surveillance management (2.00 ± 0.71), medication management (1.98 ± 0.69), and reproductive health management (1.95 ± 0.77). These scores, along with detailed item-level results, are shown in Table 2.

**Table 2:** Self-management average scores of items in the scale.

Catalogue	Item	Mean ± SD
Medication Management		1.98 ± 0.69
	1. I can take my medications as prescribed	2.33 ± 1.01
	2. I know the name of the drug, the dosage, the reason for the drug, the effect of the drug, the side effects and the symptoms of the side effects	1.77 ± 0.72
	3. I will continue to use my cold medicine when I am taking It	1.78 ± 0.69
	4. I do not adjust myself because my condition gets better or worsens Whole medication	2.02 ± 0.76
	5. When I feel unwell after taking the medication, I will seek medical attention in time to adjust the medication	2.02 ± 0.78
Disease surveillance management		2.00 ± 0.71
	6. I was able to assess disease activity 1 time every 3 ~ 6 months during the stable period of disease	1.78 ± 0.82
	7. I was able to assess disease activity every 0.5 ~ 1 month during the active period	1.66 ± 0.67
	8. I am aware of and able to recognize the symptoms of recurrence (e.g., erythema, alopecia, mouth ulcers, fever, arthralgia, increased proteinuria)	1.75 ± 0.68
	9. I will actively seek medical attention when I am unwell	2.52 ± 1.13
	10. I know that the disease can affect multiple organs (e.g., kidneys, brain)	2.31 ± 0.97
Daily Life Management		2.94 ± 0.94
	11. I do not dye my hair, perm it, tattoo my eyebrows, get my nails done	3.44 ± 1.17
	12. I can go out to protect myself from the sun, especially not between 10 a.m. and 4 p.m. when the sun is strong	3.36 ± 1.19
	13. When I have questions about a disease, I take the initiative to consult a medical professional	2.96 ± 1.09
	14. I would seek out a family member or friend to supervise the management of my illness	2.93 ± 1.06
	15. I was able to avoid overexertion	2.89 ± 1.06
	16. I try to go to bed early every night	2.78 ± 1.07
	17. I do not go to crowded places during flu season	2.76 ± 1.07
	18. I can do it with the heart of my heart	2.72 ± 1.04
	19. I believe that the disease can be controlled through active management.	2.81 ± 1.07
	20. I can avoid getting emotional	2.72 ± 1.05
Reproductive Health Management		1.95 ± 0.77
	21. I can take medication as prescribed by my doctor during pregnancy, pregnancy, and breastfeeding	1.77 ± 0.77
	22. I was able to choose how I was fed, as I was told	1.78 ± 0.79
	23. I know that women of childbearing age need to use highly effective contraception	1.87 ± 0.79
	24. I was able to have a preconception consultation and assessment when I wanted to get pregnant	2.19 ± 1.01
	25. I was able to follow up with my doctor on time when I was pregnant	2.14 ± 0.95

Among individual items, “I do not dye my hair, perm it, tattoo my eyebrows, get my nails done” received the highest score (3.44 ± 1.17), while “I was able to assess disease activity every 0.5 ~ 1 month during the active period” scored the lowest (1.66 ± 0.67). Complete item-level scores are shown in Table 2.

### Comparison of self-management scores of SLE patients with different characteristics

Table 3 shows the comparison results of SLE patients’ self-management scores with different demographic characteristics, and the results showed that age, complications, disease activity, educational level, marital status, per capita monthly income, type of medical insurance, place of residence and SLE self-management scores were correlated (*p* < 0.05).

**Table 3:** Comparison of self-management scores among SLE patients with different characteristics.

Items	Self-management score of SLE patients (x ± s)	*Z*/*H*	*p*
Gender		−0.761	0.447
Male	57.50 ± 14.03		
Female	59.21 ± 16.99		
Age(years)		30.353	<0.001*
18–30	56.38 ± 17.13		
31–40	65.28 ± 15.07		
41–60	55.13 ± 15.26		
>60	52.45 ± 20.60		
Duration of illness(years)		5.660	0.059
<5	57.97 ± 16.80		
5–10	62.41 ± 17.31		
>10	63.90 ± 11.90		
Complications		−2.275	0.023*
No	56.16 ± 16.70		
Yes	60.39 ± 16.63		
Disease Activity		12.271	0.002*
Basically, no activity	62.22 ± 16.20		
Light activity	59.66 ± 16.24		
Moderate-to-high activity	54.95 ± 17.11		
Work situation		1.517	0.468
Employed	58.29 ± 16.63		
Unemployed	60.25 ± 16.53		
Freelancing	58.08 ± 17.15		
Educational level		13.037	0.011*
Elementary school and below	55.11 ± 18.58		
Junior high school	56.08 ± 16.97		
High school or vocational school	87.33 ± 11.05		
Junior college	58.94 ± 16.70		
Bachelor’s degree or above	60.91 ± 14.69		
Marital status		6.652	0.036*
Married	60.62 ± 16.75		
Unmarried	55.34 ± 17.07		
Others	58.37 ± 14.74		
Monthly household income (RMB, Yuan)		15.064	0.005*
≤1,000	59.24 ± 17.40		
1,000–1999	59.30 ± 15.64		
2000–2,999	54.22 ± 18.26		
3,000–3,999	56.56 ± 17.09		
≥4,000	63.86 ± 14.79		
Healthcare insurance methods		14.366	<0.001*
Employee Medical Insurance	56.39 ± 16.84		
Resident Medical Insurance	60.65 ± 16.30		
Others	66.05 ± 15.32		
Family residence		10.502	0.005*
City	61.20 ± 15.93		
Rural	54.21 ± 17.71		
County seat	60.32 ± 16.24		
Biologics		−1.490	0.136
Used	61.17 ± 15.71		
Not used	58.12 ± 17.13		

SD, Standard Deviation; H, statistic of Kruskal–Wallis Test; *Z*, statistic of Mann–Whitney U test; *Significant association (*p*-value < 0.05).

### Spearman correlations between the study variables

Before presenting the correlations, a brief descriptive summary of the results from the various scales used in this study is provided:

#### Chronic Disease Self-Efficacy Scale

This scale accesses patients’ confidence in their ability to manage chronic diseases. A moderate positive correlation was observed between the self-management scores and chronic disease self-efficacy among SLE patients (*r* = 0.259, *p* < 0.001), suggesting that higher self-efficacy is associated with better self-management.

#### Brief Illness Perception Questionnaire

This scale evaluates the patient’s perception of their illness. A negative correlation between the self-management score and illness perception was observed (*r* = −0.320, *p* < 0.001), indicating that patients who perceive their illness more negatively tend to have poorer self-management.

#### Medical Coping Styles Questionnaire

This scale assesses coping styles, including confrontation, avoidance, and submission. A positive correlation was found between self-management and confrontation (*r* = 0.291, *p* < 0.001), suggesting that active confrontation of illness challenges is linked to better self-management. In contrast, avoidance (*r* = −0.195, *p* < 0.001) and submission (*r* = −0.204, p < 0.001) were negatively correlated with self-management, indicating that these coping styles are associated with poorer self-management.

#### Family Apgar Index

This index measures family functioning, including emotional support and care. A strong positive correlation was found between the Family Apgar Index and self-management (*r* = 0.553, *p* < 0.001), suggesting that better family support is linked to improved self-management.

#### Social Support Rate Scale

This scale measures the level of social support a patient receives. A moderate positive correlation was observed between social support and self-management (*r* = 0.234, *p* < 0.001), indicating that greater social support is associated with better self-management (see Table 4).

**Table 4:** Spearman correlations between the study variables.

N = 370	Spearman correlation	*p*
Chronic Disease Self-Efficacy	0.259	<0.001*
Brief Illness Perception	−0.320	<0.001*
Medical Coping Styles		
Confront	0.291	<0.001*
Avoid	−0.195	<0.001*
Succumb	−0.204	<0.001*
Family Apgar Index	0.553	<0.001*
Social Support Rate	0.234	<0.001*

Chronic Disease Self-Efficacy: Chronic Disease Self-Efficacy Scale. Brief Illness Perception: Brief Illness Perception Questionnaire. Medical Coping Styles: Medical Coping Styles Questionnaire. Confront: Subscale of Medical Coping Styles (Confrontation). Avoid: Subscale of Medical Coping Styles (Avoidance). Succumb: Subscale of Medical Coping Styles (Submission). Family Apgar Index: Family Apgar Index. Social Support Rate: Social Support Rate Scale.” *Significant association (*p*-value < 0.05).

### Multivariate analysis of SLE self-management scores

The self-management ability scores of SLE patients were used as the dependent variable. Independent variables included age, complications, disease activity, educational level, marital status, monthly household income, healthcare insurance methods, family residence, and various psychological and social factors. These factors involved chronic disease self-efficacy, brief illness perception, medical coping styles (including confrontation, avoidance, and submission), Family Apgar Index, and social support rate. The assignment methods for these independent variables are shown in Table 5. Since all variance inflation factors (VIFs) were below three and tolerance values were above 0.4, multicollinearity was not a concern. The results indicated that disease activity, educational level, marital status (unmarried), chronic disease self-efficacy, brief illness perception, medical coping styles (confrontation, avoidance, and submission), Family Apgar Index, and social support rate were significant predictors of SLE self-management (*p* < 0.05). Additionally, the multiple linear regression model explained 52.4% of the variance in SLE self-management (adjusted *R*^2^ = 0.524, *p* < 0.001), as shown in Table 6.

**Table 5:** Assignment of independent variables.

Variable	Assignment of value
Age(years)	18–30 = 1, 31–40 = 2, 41–60 = 3, >60 = 4
Complications	No = 0, Yes = 1
Disease activity	Basically, no activity = 1, Light activity = 2, Moderate-to-high activity = 3
Educational level	Elementary school and below = 1, Junior high school = 2, High school or vocational school = 3, Junior college = 4, Bachelor’s degree or above = 5
Monthly household income (RMB, Yuan)	≤1,000 = 1, 1,000–1999 = 2, 2000–2,999 = 3, 3,000–3,999 = 4, ≥ 4,000 = 5
Marital status	Using married as a reference, set 2 dummy variables
Healthcare insurance methods	Taking the employee medical insurance as a reference, two dummy variables were set
Family residence	Using the city as a reference, set 2 dummy variables
Chronic disease self-efficacy	Substitute the original value
Brief illness perception	Substitute the original value
Medical coping styles	
Confront	Substitute the original value
Avoid	Substitute the original value
Succumb	Substitute the original value
Family Apgar Index	Substitute the original value
Social support rate	Substitute the original value

**Table 6:** Multivariate analysis of self-management in SLE patients.

Variable	*B*	SE	Beta	*t*	*p*	95%CI
Constant	45.424	8.813		5.154	<0.001*	28.091–62.757
Age(years)	−1.526	0.882	−0.081	−1.729	0.085	−3.262–0.209
Disease activity	−1.938	0.785	−0.096	−2.470	0.014*	−3.482 to −0.395
Educational level	1.346	0.619	0.111	2.174	0.030*	0.128–2.564
Monthly household income (RMB, Yuan)	−0.192	0.557	−0.016	−0.344	0.731	−1.288–0.904
Marital status						
Unmarried	−5.176	1.779	−0.134	−2.909	0.004*	−8.676 to −1.676
Others	−0.948	2.217	−0.017	−0.428	0.669	−5.308–3.412
Healthcare insurance methods						
Resident Medical Insurance	0.753	1.695	0.022	0.444	0.657	−2.581–4.086
Others	3.689	2.186	0.071	1.688	0.092	−0.610–7.988
Family residence						
Rural	−2.817	1.749	−0.076	−1.611	0.108	−6.256–0.623
County seat	−3.033	1.738	−0.073	−1.745	0.082	−6.450–0.385
Chronic disease self-efficacy	0.256	0.061	0.165	4.166	<0.001*	0.135–0.376
Brief illness perception	−0.252	0.060	−0.164	−4.202	<0.001*	−0.370 to −0.134
Medical coping styles						
Confront	0.783	0.170	0.186	4.605	<0.001*	0.449–1.117
Avoid	−0.654	0.183	−0.145	−3.575	<0.001*	−1.013 to −0.294
Succumb	−0.836	0.380	−0.090	−2.199	0.029*	−1.584 to −0.088
Family Apgar Index	2.950	0.305	0.393	9.672	<0.001*	2.350–3.550
Social support rate	0.193	0.078	0.096	2.484	0.013*	0.040–0.346

B, Unstandardized regression coefficient; SE, Standard error of the regression coefficient; Beta, Standardized regression coefficient (measures the relative importance of predictors); *t*, *t*-statistic for testing if the regression coefficient is significantly different from zero; *p*, *p*-value for testing the statistical significance of the coefficient (values < 0.05 are considered statistically significant); 95% CI, 95% Confidence Interval for the regression coefficient (range of values in which the true population coefficient is likely to fall). *Significant at *p* < 0.05.

## Discussion

The results of this study revealed that the self-management score of SLE patients was 59.06 ± 16.75. This indicates a moderate level of self-management ability (11, 14), demonstrating that SLE patients’ ability to manage their condition requires improvement. Higher self-management scores reflect better ability in managing the disease (14), and in this cohort, a score of 59.06 indicates that there is room for improvement in self-care practices. However, it is challenging to directly compare these results with previous studies, as few have utilized the same measurement tools employed in this study. In terms of specific dimensions, the mean score for daily life management was the highest (2.94 ± 0.94), while the mean score for reproductive health management was the lowest (1.95 ± 0.77). This suggests that SLE patients demonstrated stronger self-management in daily life activities, while facing significant challenges in managing reproductive health. The difficulty in reproductive health management may be attributed to the high proportion of women of childbearing age among SLE patients (29). These women often lack sufficient knowledge about reproductive health and harbor concerns about the impact of medications on fertility, as well as fears of poorly controlled SLE and adverse pregnancy outcomes (30). Similar findings have been reported in other studies, where SLE patients showed particular difficulty in reproductive health management due to these concerns and the complexities of managing both the disease and pregnancy (29–31). It is, therefore, recommended that more attention be given to the reproductive health needs of SLE patients, particularly for women of childbearing age. Health professionals should provide timely, targeted prenatal counseling and adopt a multidisciplinary approach to support SLE patients in managing their reproductive health (4). This may help reduce the risk of pregnancy complications and improve the overall quality of life for these patients.

This study used the socioecological model as a theoretical framework to reveal the impact of multi-level factors on patients’ self-management ability. The results showed that disease activity, educational level, unmarried status, chronic disease self-efficacy, disease perception, medical coping style, family support and social support and other factors significantly affected the self-management behavior of SLE patients from the micro, meso, and macro levels.

At the micro-system level, we found that disease activity was significantly negatively correlated with self-management ability in SLE patients, and patients with high disease activity tended to exhibit lower self-management ability. High disease activity not only results in physical discomfort but also affects the mood and cognition, limiting the patient’s ability to self-manage (32). The results of this study suggest that patients with higher literacy tend to have stronger self-management skills, consistent with other studies (31–33). This may be because patients with higher levels of education have greater access to disease-related information, are more aware of their illness and health management, and are more active in discussing their condition and treatment plans with healthcare professionals (32). Unmarried patients with SLE have lower self-management skills, which may be because unmarried patients are lonelier than married patients, lack the support and care of their spouses, and are more likely to face difficulties in disease management and experience psychological distress, which in turn affects their ability to manage themselves (34). Caregivers can adopt modular health education programs to design different learning content for patients with varying levels of education and marital status, help patients quickly acquire disease-related knowledge, regularly assess their knowledge mastery, and adjust the education content according to feedback to ensure the effectiveness of education (35), thereby improving self-management ability and reducing disease activity.

Self-efficacy also falls under the microsystem level, and this study found that the higher the level of self-efficacy, the greater the patients’ skill in managing chronic diseases. Self-efficacy refers to an individual’s perception of effective action in various situations, which directly affects patients’ confidence in their ability to manage their own diseases and is an important factor in patients’ self-management (35). Studies have shown that patients with high self-efficacy are more willing to engage in positive self-management behaviors and are better able to cope with the challenges posed by the disease (36). Therefore, clinical nurses should focus on improving patients’ self-efficacy and enhancing their confidence in their own disease management by setting small goals, sharing successful experiences, providing positive feedback and support, etc., thereby increasing their self-management enthusiasm and persistence (37).

This study found that disease perception and medical coping methods also affect the self-management abilities of SLE patients at the micro-level, consistent with previous studies (38, 39). The more positive the patient’s awareness of the disease, the more confident he is in managing the disease (38). Conversely, patients with poor perception of the consequences of illness have a poorer sense of identification with the disease, a more negative psychological state, lower expectations of treatment, and a poorer level of self-management (39). The coping dimension was positively correlated with self-management behavior among SLE patients, whereas the avoidance and submission dimensions were negatively correlated. This may be because the patients who adopt this aggressive coping style are more willing to communicate with healthcare providers, actively seek external support, and actively participate in disease management (40). Relevant studies have confirmed that patients’ risk perception and coping styles are closely related to self-management behaviors (38), so it is recommended that medical staff pay attention to reducing the output of invalid information when educating them, so as not to increase patients’ anxiety, guide patients correctly, enhance their confidence in managing diseases, and help patients adjust their emotional states promptly, also encouraging them to face the disease with a positive attitude and then improve their self-management behaviors (39–41).

At the mesosystem level, this study found that the greater the level of family care for SLE patients, the higher their self-management ability. Family care reflects family members’ subjective satisfaction with family functioning, and studies have shown that good family functioning improves patients’ self-management behaviors and treatment adherence (41). According to the socio-ecological system theory (15–17), an individual’s health behavior is influenced by their intrinsic psychological factors. Care and support from family members can enhance patients’ self-efficacy, making them more confident and optimistic in the face of illness and more effective in self-management (40). It is recommended that clinical medical staff start with family intimacy and affection, and strengthen education for family members, so that they can master disease knowledge and urge patients to take medication on time, have regular reexaminations, and recognize symptoms of recurrence in time (42). At the same time, spouses and other family members are encouraged to take the initiative to care for patients, understand patients’ needs promptly, actively guide patients in a reasonable manner, and build their confidence in facing the disease (43).

This study found that social support was positively correlated with SLE self-management ability. Social support, as a macro-system within socio-ecological systems, not only influences behavior at the individual and community levels but also profoundly impacts patients’ health behaviors through policy and cultural climate (14–16). This suggests that the management of chronic diseases, such as SLE, should be further improved, including medical insurance coverage, patient education, and chronic disease management programs (14). Through policy support, provide a more comprehensive social support network for patients, and foster a social atmosphere that actively supports their self-management of chronic diseases through media, community activities, and public education, thereby improving their self-management skills (15). Second, it can strengthen people’s social awareness and support for SLE, and reduce discrimination and prejudice against patients (16).

This study has several notable strengths: Firstly, the sample size of 370 SLE patients ensures robust findings and greater statistical power (44). Secondly, the study employed validated instruments to assess various dimensions of self-management, including the Chronic Disease Self-Efficacy Scale, the Brief Illness Perception Questionnaire, and others, thereby enhancing the reliability and validity of the results (45). Additionally, the multi-center design of the study allows for a broader, more diverse sample, contributing to the generalizability of the findings to a wider population of SLE patients (46). Finally, the usage of the socioecological model provides a comprehensive framework that allows for the consideration of multiple influencing factors at various levels, adding depth to the understanding of self-management behaviors (47). Additionally, the multi-center design of the study allows for a broader, more diverse sample, contributing to the generalizability of the finding to a wider population of SLE patients (48). Lastly, the use of the socioecological model provides a comprehensive framework that allows for the consideration of multiple influencing factors at various levels, adding depth to the understanding of self-management behaviors (49). The findings of this study highlight the need for a targeted approach to improve self-management in SLE patients, particularly in reproductive health and disease management. The results demonstrated that factors, such as disease activity, self-efficacy, educational level, and social support, play critical roles in promoting patients’ self-management abilities. These insights have significant implications for clinical practice and patient care. Healthcare providers should consider these factors when designing interventions to improve self-management, with particular emphasis on education, psychosocial support, and reproductive health counseling. Future research should investigate these findings across diverse populations and settings, particularly through longitudinal studies, to gain deeper insights into the causal relationships and long-term effects of self-management interventions.

### Research limitations

This study has several limitations: First, the reliability and validity of the SLE self-administration scale used in this study have been established only in Chinese patients. Second, the sampling method used was convenience sampling. The selected sample size is limited, and the generalizability of the research results may be affected, so it is necessary to conduct cross-regional, large-sample, and random-sampling studies in the future. Finally, this study is cross-sectional, so it cannot infer causality.

## Conclusion

In conclusion, SLE patients’ self-management needs improvement, and clinical care managers should address their specific needs and formulate targeted interventions. By improving patients’ chronic disease self-efficacy, optimizing disease perception, promoting the use of positive coping strategies, enhancing social and family support, and improving self-management, the disease burden can be reduced and the quality of life can be improved.

## Data Availability

The original contributions presented in the study are included in the article/supplementary material, further inquiries can be directed to the corresponding author.
